# HspB2/Myotonic Dystrophy Protein Kinase Binding Protein (MKBP) as a Novel Molecular Chaperone: Structural and Functional Aspects

**DOI:** 10.1371/journal.pone.0029810

**Published:** 2012-01-17

**Authors:** Sankaralingam Prabhu, Bakthisaran Raman, Tangirala Ramakrishna, Ch Mohan Rao

**Affiliations:** Centre for Cellular and Molecular Biology, Council of Scientific and Industrial Research, Hyderabad, India; Université Joseph Fourier, France

## Abstract

The small heat shock protein, human HspB2, also known as Myotonic Dystrophy Kinase Binding Protein (MKBP), specifically associates with and activates Myotonic Dystrophy Protein Kinase (DMPK), a serine/threonine protein kinase that plays an important role in maintaining muscle structure and function. The structure and function of HspB2 are not well understood. We have cloned and expressed the protein in *E.coli* and purified it to homogeneity. Far-UV circular dichroic spectrum of the recombinant HspB2 shows a β-sheet structure. Fluorescence spectroscopic studies show that the sole tryptophan residue at the 130^th^ position is almost completely solvent-exposed. Bis-ANS binding shows that though HspB2 exhibits accessible hydrophobic surfaces, it is significantly less than that exhibited by another well characterized small HSP, αB-crystallin. Sedimentation velocity measurements show that the protein exhibits concentration-dependent oligomerization. Fluorescence resonance energy transfer study shows that HspB2 oligomers exchange subunits. Interestingly, HspB2 exhibits target protein-dependent chaperone-like activity: it exhibits significant chaperone-like activity towards dithiothreitol (DTT)-induced aggregation of insulin and heat-induced aggregation of alcohol dehydrogenase, but only partially prevents the heat-induced aggregation of citrate synthase, co-precipitating with the target protein. It also significantly prevents the ordered amyloid fibril formation of α-synuclein. Thus, our study, for the first time, provides biophysical characterization on the structural aspects of HspB2, and shows that it exhibits target protein-dependent chaperone-like activity.

## Introduction

Myotonic Dystrophy Protein Kinase Binding Protein (MKBP) associates with and activates Myotonic Dystrophy Protein Kinase (DMPK), a serine/threonine protein kinase that plays an important role in maintaining muscle structure and function [1]. CTG expansion in the 3′-untranslated region of the DMPK gene results in retention of the transcript in the nucleus, causing decreased expression of this protein, leading to the pathology of Myotonic Dystrophy1 (DM1) [2]–[4]. The selective up-regulation of MKBP observed in DM patients is believed to be a feedback mechanism to compensate for the decreased levels of DMPK [1]. The sequence of MKBP was found to be identical to that of HspB2, a member of the small heat shock protein (sHsp) family. MKBP/HspB2 gene was found to be closely linked to the αB-crystallin gene [5].

Earlier studies implicate an important role for HspB2 in cellular function and pathology. HspB2 was shown to be expressed in the heart and skeletal muscle [6]–[8]. During the differentiation of C2C12 mouse myoblast cell line, the expression of HspB2 occurs 24 hours after initiation of differentiation [9]. Although HspB2 is shown not to be heat-inducible [6], during development, it was found to be transiently up-regulated in the neonatal myocardium; it was shown to partition into the myofibrillar fraction upon heat shock [10]. It translocates from the cytosolic to the myofibril fraction of rat heart during ischemic stress [11]. HspB2 is also known to protect myocardium from ischemia and help in recovering from ischemic stress during reperfusion. It is important in ATP turnover in mouse heart [12]. HspB2 is known to be associated with mitochondria [13]. HspB2 has also been found in the senile plaques of Alzheimer disease [14]. Though these studies clearly indicate the importance of HspB2 in various cellular functions and pathology, its structural and functional aspects have not yet been addressed. We have cloned and expressed human HspB2 in *E. coli* and purified it to homogeneity. Our study for the first time sheds light on the structural aspects of HspB2 and shows that it exhibits molecular chaperone property in preventing amorphous as well as amyloid aggregation of proteins. However, unlike other mammalian sHsps characterized so far, it exhibits unique target protein-dependent chaperone activity.

## Materials and Methods

### Materials

Citrate synthase, insulin, yeast alcohol dehydrogenase and bis-ANS (bis-8-anilinonaphthalene-1-sulfonic acid) were purchased from Sigma (St. Louis, MO, U.S.A.). Phenyl Sepharose 6 fast flow, Superose 6 HR pre-packed gel filtration column and high molecular mass gel filtration calibration kit, were obtained from Amersham Pharmacia (Uppsala, Sweden). DTT was obtained from Sisco Research Laboratories (Mumbai, India). TRIzol® was supplied by GibcoBRL (Grand Island, NY, U.S.A). Advantage® RT for PCR kit was procured from BD Biosciences (Palo Alto, CA, U.S.A.). Human skeletal muscle myoblast (HSMM) and Skeletal muscle growth medium (SKGM TM-2) bullet kit were purchased from Lonza, Rockland, USA.

### Cloning of human HspB2

HSMM were maintained in SKGM TM-2 and induced to differentiate in low serum medium. Total RNA was isolated from myotubes using TRIzol® reagent and cDNA synthesized using Advantage® RT for PCR kit. Human HSPB2 was amplified using the forward primer 5′ TTA **CAT ATG** TCG GGC CGC TCA GTG -3′ (NdeI site underlined) and reverse primer 5′ CAA **AAG CTT** TCA GGG CTC AAC TAT GG -3′(HindIII site underlined). The amplicon thus obtained was cloned into pET21a expression vector employing the restriction sites in the primers and the vector. The sequence of the ligated amplicon was verified by sequencing and was found to be identical to that reported earlier.

### Expression and purification of recombinant human HspB2

The vector carrying human HspB2 sequence was transformed into *E.coli* BL21 DE3 strain (Novagen, Madison, WI, U.S.A.). The cells were cultured in L-B medium at 37°C in an orbital shaker incubator at 250 rev/min. Expression was induced by the addition of IPTG to a final concentration of 1 mM. The cells were harvested 4 hours after induction and stored at −20°C for further use. The cells were thawed and lysed by sonication in TNE buffer (50 mM Tris HCl buffer, pH 7.4, 1 mM EDTA and 100 mM NaCl) containing the protease inhibitor, PMSF (1 mM) and lysozyme (200 µg/ml).The lysate was centrifuged at 10000 *g* at 4°C for 30 min and the supernatant was subjected to ammonium sulfate precipitation. The protein precipitated out at 20% ammonium sulfate saturation. The precipitate was resolubilized in TNE buffer and applied directly onto a Phenyl Sepharose 6 Fast Flow column previously equilibrated with TNE buffer. The column was washed with three bed volumes of TNE buffer followed by elution with TNE buffer containing 40% ethylene glycol. The protein eluted out with 40% ethylene glycol. Ethylene glycol was removed by extensive dialysis against TNE buffer, pH 7.4. The protein was stored at 4°C until further use. The protein thus purified was homogeneous as determined by SDS-polyacrylamide gel electrophoresis.

### CD spectroscopy

CD spectra were recorded using a Jasco J-715 spectropolarimeter. Protein concentrations of 0.2 mg/ml (in 20 mM HEPES – NaOH buffer, pH 7.4) and 1.5 mg/ml (in TNE buffer) were used for recording far- and near-UV CD spectra in 0.1 and 1 cm path length cuvettes respectively. Each spectrum was an average of four accumulations.

### Fluorescence studies

All fluorescence studies were carried out on a Hitachi F4500 fluorescence spectrophotometer. A protein concentration of 0.2 mg/ml in TNE buffer was used for recording intrinsic tryptophan fluorescence. The measurements were made at an excitation wavelength of 295 nm and the emission spectra recorded with excitation and emission band passes set to 2.5 nm.

The hydrophobic probe, bis-ANS, was used for studying exposed hydrophobic sites. The probe was used at a final concentration of 10 µM and the protein concentration was 0.2 mg/ml (in TNE). The measurements were made at an excitation wavelength of 390 nm and excitation and emission band passes were set to 2.5 nm. Bis-ANS titration study was carried out by sequential addition of bis-ANS to the protein solution and fluorescence spectra recorded with above mentioned instrument parameters. The fluorescence intensity was plotted as a function of bis-ANS concentration. A similar experiment was also performed with αB-crystallin under identical conditions for comparison. All spectra were recorded in a corrected spectrum mode.

### Fluorescence quenching studies

Accessibility of tryptophan was analyzed by fluorescence quenching experiments. Quenching studies were performed using a Hitachi F4500 fluorescence spectrophotometer with the excitation wavelength set at 295 nm and fluorescence emission spectra were scanned from 300 to 400 nm. Fluorescence quenching titration with KI was performed at room temperature by sequentially adding aliquots of concentrated KI solution (7 M) to a 0.2 mg/ml HspB2 solution. Sodium thiosulphate was added to KI stock solution to prevent I_3_ ¯ formation.

The fluorescence quenching data in the presence of KI were analyzed by fitting to the Stern-Volmer equation:
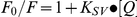
Where F_0_ and F are fluorescence intensities in the absence or presence of KI, respectively, K_SV_ is the Stern-Volmer quenching constant and Q is the KI concentration. Percentage accessibility of tryptophan to quencher was calculated using Lehrer plot [15]. Similar studies were also carried out with N-acetyl tryptophanamide (NATA), a modified amino acid tryptophan under identical conditions.

*Sedimentation velocity measurements*- Sedimentation velocity measurements were performed using an Optima XL-I analytical ultracentrifuge (Beckman Coulter, Fullerton, CA, USA). Samples of 0.125, 1 and 3 mg/ml of HspB2 in TNE buffer were subjected to centrifugation at 40,000 rpm at 20°C. The protein boundary was scanned at 1 min intervals using interference optics. The sedimentation coefficient S_20,w_ and molecular mass of the protein was calculated using the program SEDFIT [16] which uses non-linear regression fitting of the sedimenting boundary profile with Lamm equation,
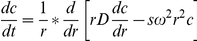
which describes the concentration distribution c(r, t) of a species with sedimentation coefficient s and diffusion coefficient D in a sector-shaped volume and in the centrifugal field ω^2^r.

### Chaperone-like Activity

The chaperone-like activity of HspB2 was assayed by monitoring the DTT-induced aggregation of insulin in 10 mM phosphate buffer (pH 7.2) containing 100 mM NaCl at 37°C either in the absence or in the presence of HSPB2. Buffer containing HSPB2 at different concentrations was incubated at 37°C for 5 min before addition of insulin to a final concentration of 0.2 mg/ml with constant stirring in a cuvette. Temperature was maintained at 37°C using a Julabo thermostatic water bath. The actual temperature in the cuvette was determined using a Physitemp microthermocouple thermometer system. Aggregation of insulin was initiated by adding 24 µl of 1 M DTT to a final assay volume of 1.2 ml. The extent of aggregation was monitored by measuring the light scattering at 90° in a Hitachi F4000 fluorescence spectrophotometer with both excitation and emission wavelengths set to 465 nm and band passes set to 3 nm.

Thermal aggregation of citrate synthase was carried out in 40 mM HEPES-KOH buffer (pH-7.5) at 43°C. The buffer containing different concentrations of HspB2 was incubated at 43°C for 8 min before the addition of citrate synthase to a final concentration of 25 µg/ml in an assay volume of 1.2 ml. Aggregation was monitored by light scattering as described above.

The thermal aggregation of yeast alcohol dehydrogenase in 50 mM phosphate buffer, pH 7.5, containing 100 mM NaCl was carried out at 48°C. Buffer containing the indicated concentrations of HspB2 was incubated at 48°C for 8 min before the addition of yeast alcohol dehydrogenase to a final concentration of 0.2 mg/ml. Aggregation was initiated by the addition of 2 mM EDTA. Aggregation was monitored by measuring light scattering as described above.

### Subunit exchange studies using fluorescence resonance energy transfer (FRET)

Subunit exchange studies were performed essentially as described for αA-crystallin earlier [17], [18]. The sole cysteine residue of HspB2 was covalently labeled with the fluorescence probes 4-acetamido-4′-((iodoacetyl)amino)stilbene-2,2′-disulfonic acid (AIAS) and Lucifer Yellow Iodoacetamide (LYI) separately by incubating the protein (1 mg/ml) in 20 mM HEPES buffer (pH 7.4) containing 100 mM NaCl with 4 mM of each of the probes for at least 18 hrs at 37°C. Following incubation, the labeled protein was separated from unreacted probes by passing the solution through a desalting column (PD10) equilibrated with 20 mM HEPES buffer (pH 7.4) containing 100 mM NaCl. Percentage labeling for each of the probes was estimated by molar absorption coefficient of the probes.

Subunit exchange was studied by mixing the AIAS- and LYI-labeled proteins in equimolar ratios in 20 mM HEPES buffer (pH 7.4) containing 100 mM NaCl at 4, 20 or 37°C. A small aliquot (10 µl) was withdrawn at different intervals of time and diluted to 500 µl with 20 mM HEPES buffer (pH 7.4) containing 100 mM NaCl. Fluorescence spectra were recorded from 350 to 600 nm in Hitachi F-4500 fluorescence spectrophotometer with the excitation wavelength set at 332 nm. The excitation and emission band passes were set to 5 nm each.

### Amyloid fibril growth of α-synuclein

Monomeric α-synuclein (1 mg/ml) in 20 mM HEPES buffer (pH 7.0) containing 100 mM NaCl was incubated with 0.15 mM SDS with vigorous stirring at 37°C overnight. Fibrils of α-synuclein so obtained were sonicated at amplitude of 20 with 5 second on/off cycle in a Sonics vibracell sonicator. The sonicated fibril sample was used as seed for initiating seeded amyloid growth.

To study the effect of HspB2 on the seeded fibril growth of α-synuclein, the sonicated fibril seed of α-synuclein (10% w/w) was added to 0.5 mg/ml α-synuclein in the absence or the presence of various concentrations of HspB2 in 20 mM HEPES buffer (pH 7.0) containing 100 mM NaCl and 0.15 mM SDS. The mixture was incubated at 37°C. An aliquot (5 µl) was drawn at various intervals and diluted to 500 µl in 50 mM glycine-NaOH buffer (pH 8.8) containing 10 µM ThT. Increase in ThT fluorescence was monitored in a Hitachi F-4000 Fluorescence spectrophotometer with the excitation and emission wavelengths set at 445 and 485 nm respectively. The excitation and emission band passes were set to 10 nm. Increase in the ThT fluorescence indicates fibril growth and is proportional to the extent of fibril formation.

## Results

**Figure 1:**
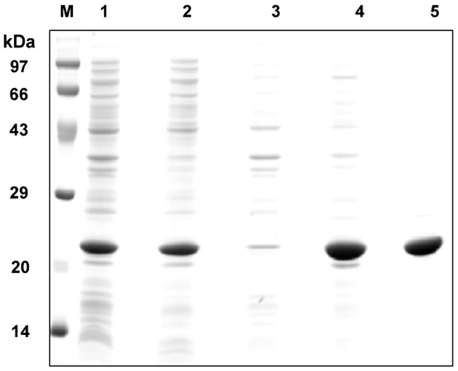
SDS-PAGE pattern showing purification of HspB2 from the soluble fraction of *E.coli* BL21(DE3) cells over-expressing human HspB2. Lane 1, whole cell lysate; Lane 2, soluble fraction; Lane 3, insoluble fraction; respectively, Lane 4, HspB2 precipitated by 20% saturated ammonium sulfate; and Lane 5, HspB2 purified by Phenyl Sepharose chromatography.

We have cloned MKBP/human HspB2 and expressed it in *E. coli*. It was obtained mainly in the soluble fraction (Figure 1). HspB2 was precipitated out with 20% saturated (NH_4_)_2_SO_4_. By a simple, one-step method of hydrophobic interaction chromatography, we could purify HspB2 in good yields and high homogeneity (Figure 1).

**Figure 2:**
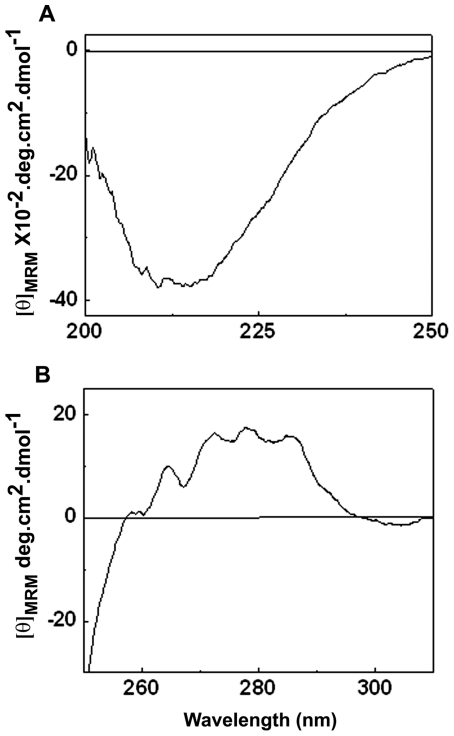
Far-UV (a) and near-UV (b) CD spectra of HspB2. Solid line, human HspB2; dotted line, αB-crystallin. The far- and near-UV CD spectra of the proteins, were recorded using 0.1 cm and 1.0 cm path length cuvettes respectively. [θ]MRW is mean residue ellipticity.

We studied the secondary structure of HspB2 by far-UV Circular Dichroism (CD) spectroscopy. The far-UV CD spectrum of HspB2 exhibits a minimum at ∼215 nm (Figure 2(a)) and represents characteristic features indicating that the protein has significant β-sheet structural elements. Analysis of the CD spectrum using the CDNN program shows that the protein contains 3.4% helix, 50.5% β-sheet and 53.9% random coil.

**Figure 3:**
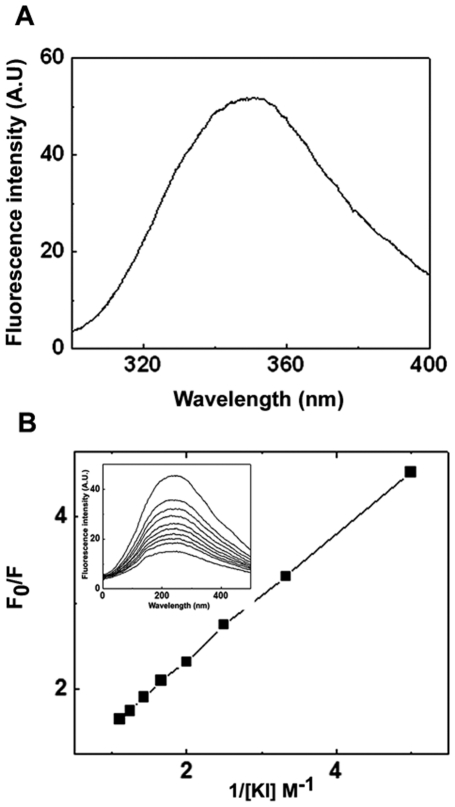
(a) Intrinsic fluorescence spectrum of HspB2. The excitation wavelength was 295 nm. The excitation and the emission band passes were set at 2.5 nm. The concentration of the proteins was 0.2 mg/ml. Fluorescence intensity is represented in arbitrary units. (b) Lehrer plot of tryptophan fluorescence quenching of HspB2 by KI. Fluorescence was monitored after sequential addition of KI to a solution containing 0.2 mg/ml HspB2. Insert shows the decrease in fluorescence intensity upon sequential addition of KI.

The tertiary structure of HspB2 was investigated by near-UV CD spectroscopy. Figure 2(b) shows that the near-UV CD spectrum of HspB2 exhibits chirality in the region between 250–280 nm representing the chiral structure of phenylalanine and tyrosine residues and at 286 nm representing tryptophan residue, indicating a compact tertiary structure of HspB2. We further probed the tertiary structural aspects around its single tryptophan residue (at the 130^th^ position) by fluorescence spectroscopy. The intrinsic tryptophan fluorescence spectrum of HspB2 shows an emission maximum at 348 nm (Figure 3(a)), indicating that the sole tryptophan residue of HspB2 is exposed to the solvent. We have measured the accessibility of the tryptophan residue by fluorescence quenching by KI (Figure 3(b)). From the Lehrer plot [15] shown in Figure 3(b), the fractional accessibility of the Trp residue obtained was 0.8, indicating that the tryptophan residue is highly accessible to the quencher.

**Figure 4:**
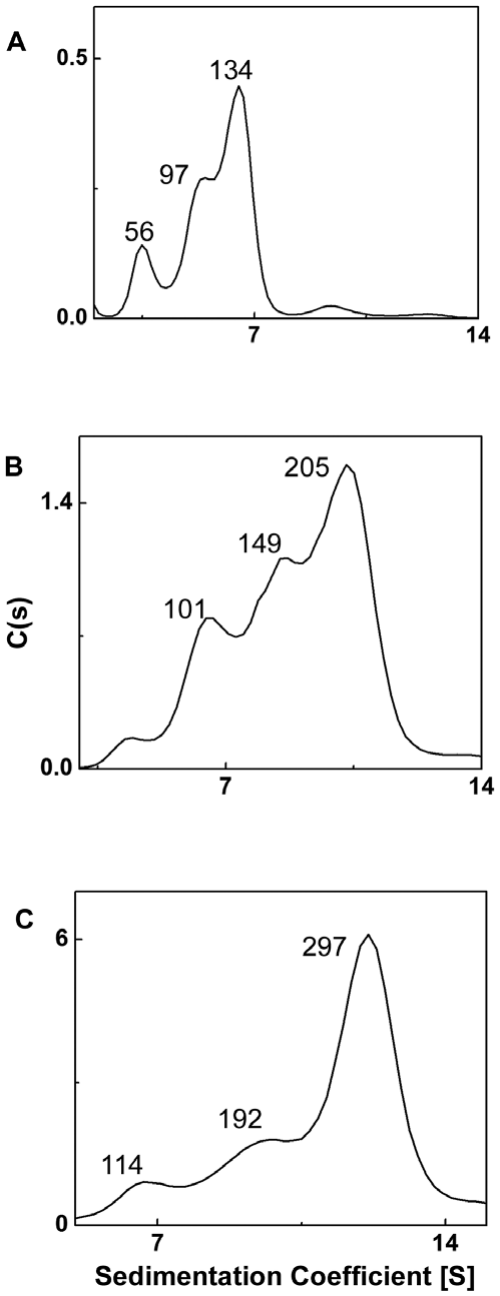
Sedimentation velocity profile of HspB2. Distribution of sedimentation coefficient of different oligomeric species changes with increasing concentration of HspB2. Panels A, B and C correspond to 0.125, 1 and 3 mg/ml of HSPB2. The numbers depicted above the peaks correspond to molecular masses (in kDa) of the oligomeric species.

In order to understand the quaternary structure of HspB2, we have performed sedimentation velocity experiment using analytical ultracentrifugation (Figure 4). Analysis of the sedimentation profile of the protein at 0.125 mg/ml using the program SEDFIT shows three populations of HspB2 with S_20,w_ values of 3.6, 5.2 and 6.5 S (figure 4A) corresponding to molecular masses of ∼56, 97 and 134 kDa, obtained from fitting the profiles to Lamm's equation (See Materials and Methods). However, when the experiment was performed at higher protein concentrations of 1 and 3 mg/ml the profile changed significantly. At a concentration of 1 mg/ml, the 56 kDa peak decreased and a significantly larger peak corresponding to 101 kDa was observed in addition to peaks corresponding to 149 and 205 kDa. Similarly at 3 mg/ml peaks corresponding to molecular masses of 114, 192 and 297 kDa were observed. (figure 4B&C). As the sample preparation of different protein concentration involved dilution from the stock of higher concentration of the protein, it appears that the different species reported by the sedimentation experiments may be in equilibrium. Thus, the sedimentation velocity measurement shows that HspB2 exhibits polydisperse populations of multimeric species. Interestingly, oligomer sizes and the relative concentrations of the species were concentration-dependent.

**Figure 5:**
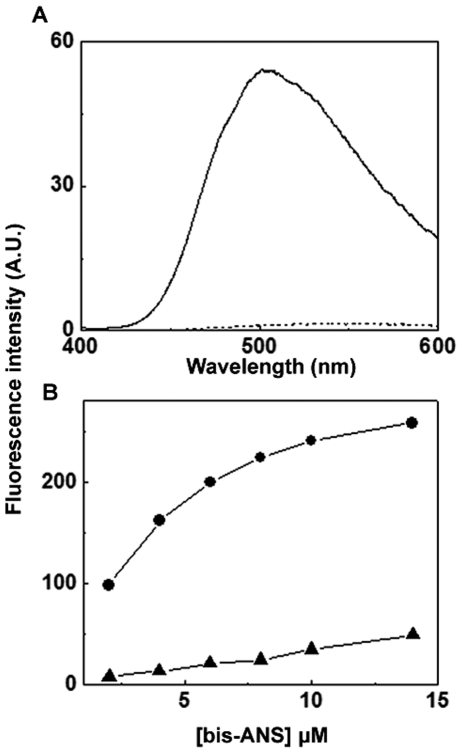
Bis-ANS binding to HspB2. (a) Fluorescence spectra of bis-ANS alone (dotted line) and bis-ANS bound to human HspB2 (solid line). Bis-ANS was added to the final concentration of 10 µM and the spectra were recorded using an excitation wavelength of 390 nm with the excitation and emission band passes set at 2.5 nm. (b) Bis-ANS titration of human HspB2. Bis-ANS fluorescence was measured as described above after sequential additions of bis-ANS to the protein solutions. (-•-) αB-crystallin, (-▴-) HspB2.

We have investigated the hydrophobic surfaces on HspB2 using the hydrophobic probe, bis-ANS. Upon binding to the hydrophobic surface of a protein, the fluorescence intensity of bis-ANS is known to increase several-fold, accompanied by a blue shift in its emission maximum [19]. Figure 5(a) shows that upon binding to HspB2 the fluorescence intensity of bis-ANS increases significantly accompanied by a shift in the emission maximum to ∼505 nm, indicating that HspB2 exhibits accessible hydrophobic surfaces. Since hydrophobic interactions are important for molecular chaperone-like activity [20]–[22], we have compared its bis-ANS binding property with that of a known molecular chaperone, αB-crystallin. The extent of binding of bis-ANS to HspB2 is significantly less compared to that in the case of αB-crystallin (Figure 5(b)). In addition, it is also seen that while αB-crystallin exhibits saturable bis-ANS binding, HspB2 binding to bis-ANS does not seem to be saturable (the fluorescence intensity gradually increases without saturation behavior even up to the bis-ANS concentration of 200 µM (data not shown)). The reason for this unsaturable binding of bis-ANS to HspB2 is not clear. The observed lack of saturation in bis-ANS binding to HspB2 could be due to bis-ANS-binding-induced changes in the conformation as observed in the case of binding of ANS to carbonic anhydrase [23]. However, the data in the presence of lower concentrations of bis-ANS (2–10 µM) suggests that HspB2 has much lesser exposed hydrophobic surfaces than αB-crystallin.

**Figure 6:**
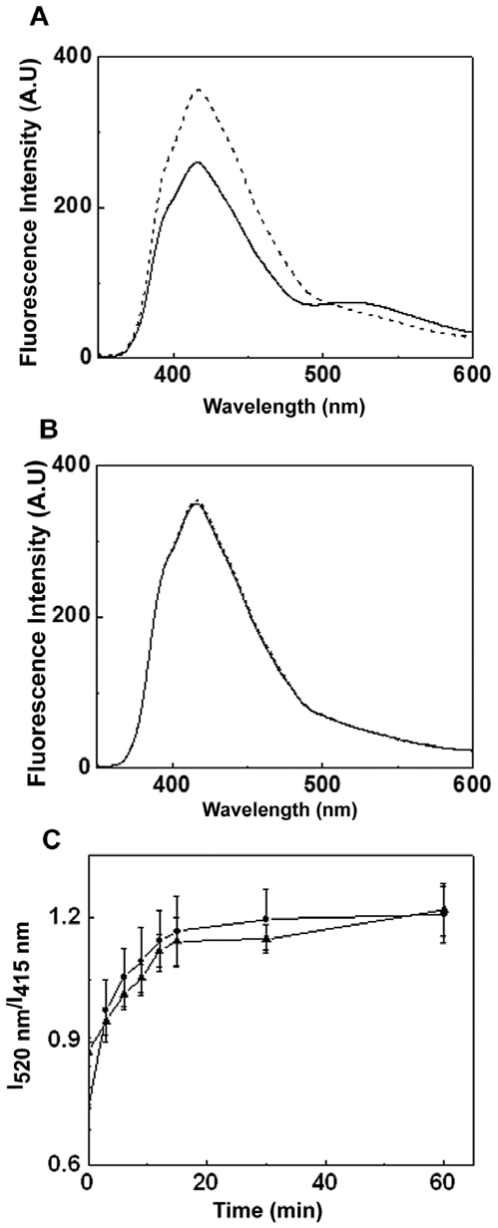
Subunit exchange studies of HspB2 at different temperatures. Subunit exchange at (a) 37°C (b) 4°C. The fluorescence spectra immediately after mixing AIAS and LYI labeled HspB2 in equimolar ratios (dotted line) and after 15 min (solid line) are represented. A decrease in AIAS fluorescence intensity with concomitant increase in LYI fluorescence intensity indicative of subunit exchange is seen when the mixture is incubated at 37°C. No such change was seen at 4°C. The rate of subunit exchange at 37°C (•), 20°C (▴) are compared (c). The rates of subunit exchange at these two temperatures are similar.

We have investigated whether HspB2 oligomers exhibit dynamic subunit exchange property. Subunit exchange has been studied in αA-crystallin by fluorescence resonance energy transfer using AIAS-labeled αA-crystallin as a donor and LYI-labeled αA-crystallin as an acceptor [17]. HspB2 has a sole cysteine residue at the 118^th^ position in its sequence. We have labeled the cysteine residue of HspB2 by AIAS and LYI and studied whether the labeled protein exhibits FRET upon mixing. Figure 6a shows a decrease in the fluorescence intensity of AIAS with some increase in the fluorescence intensity of LYI upon incubating the mixture of AIAS- and LYI-labeled HspB2 at 37°C for 15 min. This result indicates a FRET between the fluorophores and hence the subunit exchange process between the AIAS- and LYI-HspB2 oligomers. Figure 6b shows the fluorescence spectrum of the mixture of AIAS- and LYI-labelled HspB2 incubated at 4°C. The spectrum exhibits the fluorescence spectral characteristics only of AIAS without any change with respect to incubation time, indicating that HspB2 does not exhibit subunit exchange at 4°C. However, we have compared the subunit exchange process of HspB2 at 37°C and 20°C as a function of time and found that the subunit exchange kinetics are not significantly different at these two temperatures (Figure 6(c)). Thus, our study shows that HspB2 exhibits the property of subunit exchange among its oligomeric species.

**Figure 7:**
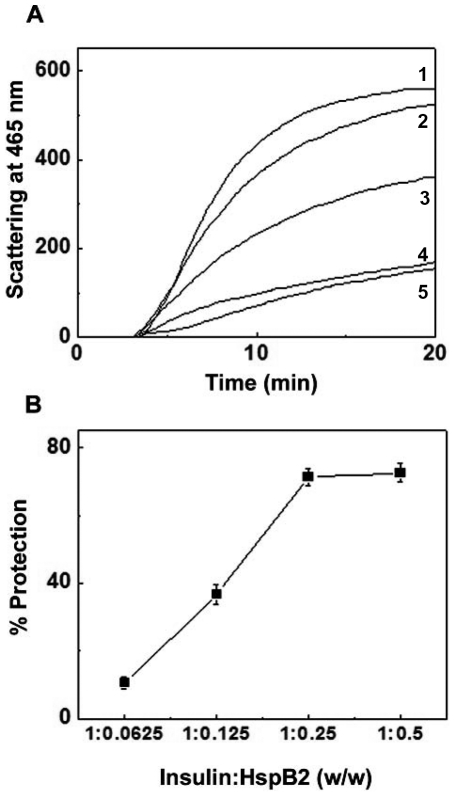
Chaperone-like activity of human HspB2 towards DTT-induced aggregation of insulin. (a) Aggregation profile of 0.2 mg/ml insulin alone (1); and in the presence of 1∶0.0625, (2); 1∶0.125, (3); 1∶0.25, (4); and 1∶0.5, (5) ratios of insulin to HspB2 (w/w). (b) The percentage protection of DTT-induced aggregation of insulin by HspB2 as a function of weight ratio of chaperone to target protein.

We have investigated whether HspB2 exhibits chaperone-like activity towards amorphous aggregation of various target proteins. Upon reduction of the disulfide bonds, the B-chain of insulin aggregates [24], leading to increase in light scattering (Figure 7(a)). In the presence of HspB2, the aggregation of insulin decreased as a function of HspB2 concentration (Figure 7(a), indicating that it exhibits chaperone-like activity against the DTT-induced aggregation of insulin. At insulin to HspB2 ratio of 1∶0.125 (w/w), HspB2 exhibited ∼37% prevention of aggregation, which increased to ∼71% at insulin to HspB2 ratio of 1: 0.25. With further increase in HspB2, the extent of protection remained at ∼72.5% (Figure 7(b)).

**Figure 8:**
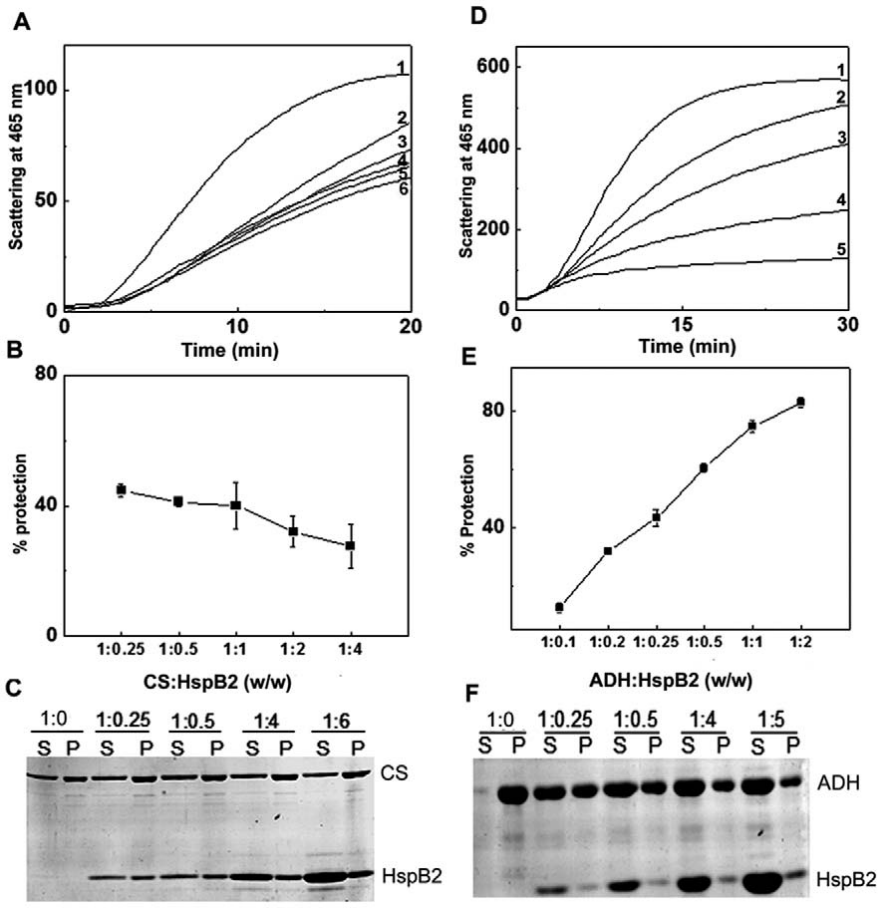
Effect of HspB2 on the thermal aggregation of citrate synthase (CS) at 43°C. (a) Aggregation profile of 25 µg/ml CS alone (1) and in the presence of 1∶4, (2); 1∶2, (3); 1∶1, (4) 1∶0.5, (5) and 1∶0.25, (6) ratios of CS to HspB2 (w/w). (b) The apparent percentage protection calculated from light scattering profile as a function of the weight ratio of CS to HspB2. (c) The SDS-PAGE pattern of soluble (S) and precipitate (P) fractions of mixtures of various citrate synthase to HspB2 ratios used in the chaperone assays. The soluble fraction and the precipitate were obtained by centrifugation at the end of the chaperone assay. (d) Effect of HspB2 on the thermal aggregation of yeast alcohol dehydrogenase (ADH) at 48°C. Aggregation profile of 0.2 mg/ml ADH alone (1) and in the presence of 1∶0.1, (2); 1∶0.2 (3); 1∶0.5, (4); 1∶2 (5); ratios of alcohol dehydrogenase to HspB2 (w/w). (e) the percentage protection calculated from light scattering profile as a function of the weight ratio of ADH to HspB2. (f) SDS-PAGE pattern of soluble (S) and precipitate (P) fractions of mixtures of various ADH to HspB2 ratios used in the chaperone assays. The soluble fraction and the precipitate were obtained by centrifugation at the end of the chaperone assay.

We have also performed the chaperone assay of HspB2 using thermal aggregation of citrate synthase at 43°C as a model system (Figure 8(a)-(c)). Surprisingly, unlike in the case of insulin, HspB2 did not exhibit concentration-dependent increase in prevention of aggregation of citrate synthase. At a citrate synthase to HspB2 ratio of 1: 0.25 (w/w), the light scattering decreased partially, indicating partial protection by HspB2. However, as the concentration of HspB2 increased, there was a gradual but slight increase in light scattering, indicating that percent protection decreased with increase in HspB2 concentration (Figure 8(a & b)). Even at a citrate synthase to HspB2 ratio of 1∶8 (w/w), the percent protection decreased (data not shown). When the assay samples were centrifuged to separate the soluble protein and precipitate and the samples analyzed on SDS-PAGE, the amount of HspB2 in the precipitate increased with increase in HspB2 concentration, showing that HspB2 co-precipitated with citrate synthase (Figure 8(c)). Thus, it appears that HspB2 is unable to effectively prevent the aggregation of citrate synthase, whereas it prevents the aggregation of insulin to the extent of ∼75%. It is possible that HspB2 is active at 37°C, the temperature at which the insulin aggregation assay was carried out, whereas it is ineffective at the elevated assay temperature of 43°C, the assay temperature for citrate synthase aggregation; hence the lack of activity against citrate synthase. We have, therefore, investigated the chaperone-like activity of HspB2 towards the aggregation of yeast alcohol dehydrogenase (ADH) at 48°C.

Figure 8(d)–(f) shows the effect of HspB2 on the aggregation of ADH. At an ADH to HspB2 ratio of 1∶0.1 (w/w), HspB2 shows ∼12.5% protection(curve 2, fig. 8(e)). As the concentration of HspB2 is increased, it shows a progressive increase in the chaperone-like activity and at an ADH to HspB2 ratio of 1∶2 (w/w), it shows ∼83% prevention of aggregation (curve 5, figure 8(e)). The samples of ADH heated at 48°C in the absence or the presence of various concentrations of HspB2 in the chaperone assay described above were centrifuged to separate the soluble protein and the precipitate. These samples were then run on SDS-PAGE. It is evident from Figure 8(f) that while almost all the ADH was obtained in the precipitate in the absence of HspB2, the amount of ADH recovered in the soluble fraction increased as the concentration of HspB2 in the assay increased. It is interesting to note that unlike in the citrate synthase protection assay, very little HspB2 was found in the precipitate even at the highest concentration of HspB2 employed in the assay (fig. 8(f)). Thus, these studies show that HspB2 can exhibit chaperone-like activity even at 48°C. HspB2 has the ability to prevent the aggregation of insulin and ADH, but not that of citrate synthase, indicating that it exhibits target protein-dependent chaperone-like activity. This property seems to be different from that of other members of the mammalian sHsp family such as αA-crystallin, αB-crystallin, Hsp27 and Hsp22 [25]–[30].

**Figure 9:**
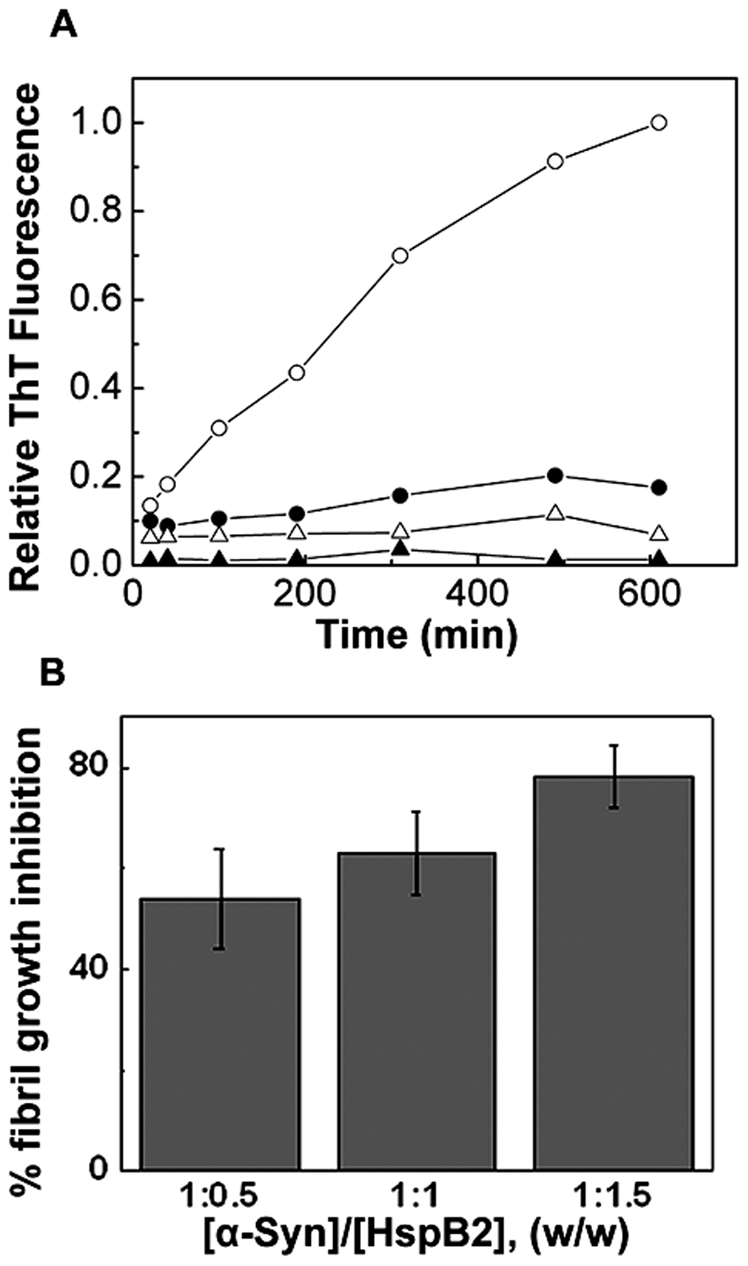
Prevention of α-synuclein amyloid fibril formation by HspB2. (a) Amyloid fibril formation of α-synuclein in the absence (-○-) and in the presence of HspB2 at α-synuclein to HspB2 ratio of 1∶1.5 (-•-) monitored by ThT fluorescence. HspB2 incubated with α-synuclein seeds (-▴-), or HspB2 alone (-Δ-) showed negligible ThT fluorescence. (b) Percentage prevention of α-synuclein amyloid fibril growth in the presence of various concentrations of HspB2. Error bars for 7 sets of experimental data are shown.

We have investigated whether HspB2 exhibits chaperone activity in preventing well-ordered amyloid fibril formation. α-Synuclein is a natively unstructured presynaptic protein whose aggregation and amyloid fibril formation is involved in some neurodegenerative diseases such as Parkinson's disease, multiple system atrophy and other synucleinopathies including dementia with Lewy bodies [31], [32]. The anionic detergent SDS, which mimics membrane environment, has been shown to promote amyloid fibril formation *in vitro* [33], [34]. We have investigated the effect of HspB2 on the SDS-induced amyloid fibril formation of α-synuclein as a model system. Figure 9(a) shows the amyloid fibril formation of α-synuclein upon seeding with the sonicated preformed fibril seed as monitored by Thioflavin-T (ThT) fluorescence. At an α-synuclein to HspB2 ratio of 1∶0.5 (w/w), HspB2 prevents the seeded amyloid fibril growth of α-synuclein to an extent of ∼54%. The extent of prevention of amyloid fibril formation increases to ∼63% and to ∼78% at 1∶1 and 1∶1.5 (w/w) ratios respectively of α-synuclein to HspB2 (Figure 9 (b). This result indicates that HspB2 exhibits chaperone property in preventing the amyloid fibril formation of α-synuclein.

**Figure 10:**
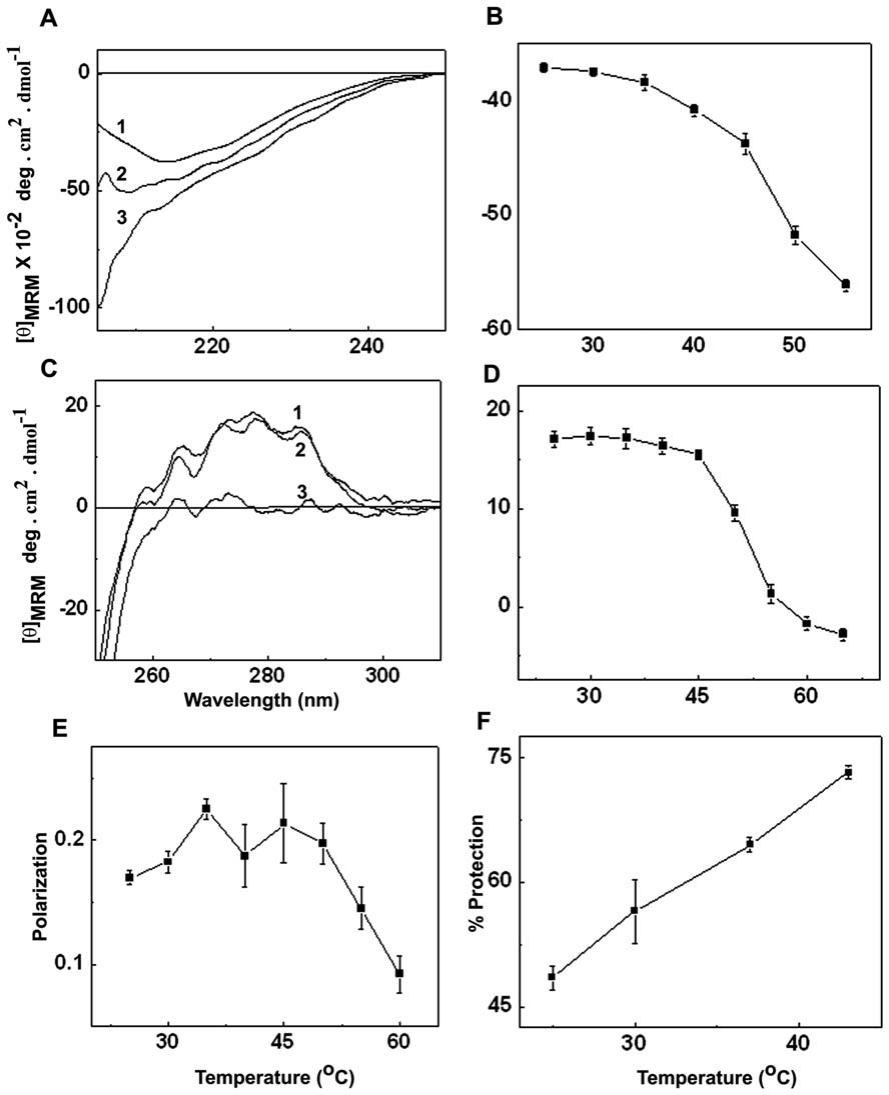
Temperature-induced changes in the conformation and chaperone property of HspB2. (A) far UV-CD spectra of HspB2 at 25°C (1), 45°C (2) and 55°C (3). (B) The change in the mean residue mass ellipticity ([θ]MRM) at 214nm as a function of temperature. (C) Near UV-CD spectra of HspB2 at 25°C (1), 45°C (2) and 55°C (3). (D) The change in the [θ]MRM at 286 nm as a function of temperature. (E) The changes in the fluorescence polarization of AIAS-labelled HspB2 with increasing temperature. (F) % Protection offered by HspB2 to the DTT-induced aggregation of insulin at different temperatures.

Interestingly, HspB2 which is predominantly present in the cytosol of cells translocates to the surface of mitochondria upon heat stress and protect cells from heat-induced cell death [13]. We have investigated temperature-induced conformational changes of HspB2, if any. Figure 10A shows the far-UV CD spectra of HspB2 at 45°C and 55°C. The overall negative ellipticity is increased, with the increase being more pronounced below 210 nm. Figure 10B shows the changes in the ellipticity at 214 nm as a function of temperature. The negative ellipticity gradually increases till 45°C and increases more sharply above this temperature. These results indicate that HspB2 does not undergo global unfolding and loss of its entire secondary structure in the temperature range studied. However, the increase in negative ellipticity which is more pronounced below 210 nm indicates a significant conformational alteration involving some local unfolding of its secondary structural elements.

Figure 10 C shows that the near-UV CD spectra of HspB2 at 25°C and at 45°C differ only marginally and hence marginal changes in its tertiary structural packing around the aromatic amino acid residues. The ellipticity at 286 nm as a function of temperature shows marginal changes till 45°C and then decreases sharply with further increase in temperature (Figure 10 (d)). We have labeled the sole cysteine residue at 118 (in the “α-crystallin domain”) with the fluorescent probe AIAS and measured its fluorescence polarization as a function of temperature (Figure 10 (e)). The polarization value increases gradually till 45°C, and decreases more sharply above this temperature resulting in the increased mobility of the probe above 45°C, indicating the increased flexibility of the region at higher temperature. Thus, all these results indicate (i) significant (local) conformational change of HspB2 around the heat shock temperatures (40–45°C) and (ii) that HspB2 loses its tertiary structure while having significant secondary structure at higher temperatures (eg. 55°C), suggesting that HspB2 exhibits a transition to a molten-globule-like state [35], [36].

Having observed temperature-dependent conformational changes in HspB2, we have investigated the effect of temperature on its chaperone-like activity towards the aggregation of insulin. Figure 10 (f) shows that the percentage protection offered by HspB2 to insulin increases significantly with increase in temperature, indicating that HspB2 exhibits higher chaperoning efficiency at temperatures relevant to heat shock.

## Discussion

MKBP/HspB2 appears to be an important player in regulating the homeostasis of the kinase, DMPK which has pleotrophic functions such as skeletal muscle integrity, cardiac muscle atrioventricular conduction and ion-channel gating to mention a few [37]. Studies on the isolated hearts from HspB2 knockout mice have shown an impaired regeneration of ATP and phosphocreatine during recovery after ischemia, indicating its central role in cardiac energetics during ischemia/reperfusion; further knockout mice that were administered dobutamine (a strong inotropic and positive chronotropic) stimulus showed that HspB2 allows for a greater capacity for increased work during acute inotropic challenge [38]. It has been reported that DMPK co-localizes with MKBP/HspB2 at the Z-discs of myocardium and also that it translocates to the myofibrillar fraction upon heat stress [9]. Despite its important role in various functions, the structural and functional aspects of HspB2 have not been understood well so far.

As mentioned earlier MKBP/HspB2 shares sequence similarity especially in the “alpha-crystallin domain” with other sHsps such as Hsp27 and alpha-crystallins. Despite sequence homology in the “alpha-crystallin domain”, members of the sHsp family differ both in length and sequence in the N-terminal domain as well as the C-terminal extension. The structural as well as functional aspects of the members could be different. A comprehensive understanding of such structural and functional differences among sHsps is not yet been addressed. Our study shows that MKBP/HspB2 exhibits several differences in structural and chaperone functional aspects from those of well-characterized Hsp27 and α-crystallins: (i) Though HspB2 contains significant β-sheet structure like other sHsps, it differs significantly in quaternary structure. Small heat shock proteins such as Hsp27, αA- and αB-crystallin are known to assemble into large oligomeric complexes with average molecular masses ranging from 400–600 kDa [39], [40]. HspB2 forms discreet sets of multimeric assemblies in a concentration-dependent manner. (ii) HspB2 exhibits subtle differences in its subunit exchange at various temperatures compared to α-crystallins. Fluorescence resonance energy transfer experiments show that HspB2 does not exhibit subunit exchange at 4°C, but exhibits comparable subunit exchange at 20 and 37°C. It has been suggested that the chaperone-like activity is correlated with the dynamics of subunit exchange [41], [42]. α-Crystallins exhibit much slower rate of subunit exchange at 20°C compared to that at 37°C [17], [43]. A structural transition above 30°C significantly increases the chaperone-like activity of α-crystallin [20], [21]. Similarly, MjHsp16.5, the small heat shock protein of *Methanococcus jannaschii* shows almost no subunit exchange at 37°C but shows temperature-induced increase in the rate of subunit exchange and increase in chaperone-like activity at temperatures above 65°C [41]. The dynamics of subunit exchange occurring at 20°C might be contributing to the significant chaperone-like activity of HspB2 against the DTT-induced aggregation of insulin at this temperature. Though the subunit exchange of HspB2 at 20°C is comparable to that at 37°C, subtle temperature-dependent conformational changes may be responsible for the observed increase in its chaperone-like activity at higher temperatures. (iii) Interestingly, unlike Hsp27 or α-crystallins, HspB2 exhibits target protein-dependent chaperone-like activity. HspB2 exhibits molecular chaperone-like activity towards the DTT-induced aggregation of insulin or heat-induced aggregation of alcohol dehydrogenase. However, it only partially prevents the heat-induced aggregation of citrate synthase, which does not improve with further increase in HspB2 concentration – in fact it co-precipitates with citrate synthase.

The extracellular presence of HspB2 has been reported in the senile plaques of Alzheimer disease and in cerebral amyloid angiopathy [14]. Our present study shows that HspB2 prevents the amyloid fibril growth of, α-synuclein which is known to be present in the plaques along with Aβ peptide. Alpha-synuclein and Aβ peptides are known to interact with each other and modulate the amyloid fibril formation [44]. Fibril fragments/nuclei of these proteins can seed the fibril formation of each other [45]. Thus, the present finding of prevention of amyloid fibril formation of alpha-synuclein by MKBP/HspB2 has physiological significance in neurodegenerative diseases.

Studies on isolated hearts of HspB2 knockout mice showed impaired systolic function and were found to be inefficient in regenerating vital molecules such as ATP and phosphocreatine during reperfusion after ischemia [12]. Another study revealed that HspB2 specifically interacts with and activates DMPK, thus ameliorating, in part, the insufficiency of DMPK molecules, due to reduced expression. It does not interact with other kinases closely related with DMPK [1]. Further, HspB2, forms a complex with HspB3, but not with other sHsps that are abundantly expressed in myocytes [9]. It appears that functions performed by HspB2 are unique and cannot be compensated by other sHsps.

There is increasing evidence that sHsps play important roles in various diversified cellular functions [46]–[48] and also are associated with several pathological conditions [49], [50]; these proteins have been proposed to have therapeutic value [51]–[53]. In this context, understanding the structural and functional properties of individual sHsps is important especially to use sHsps as therapeutic agents or targets. Our study provides the structural and chaperone functional aspects of MKBP/HspB2 and its temperature-dependent changes and highlights the differences in properties from those of other known sHsps. The findings of target protein-dependent chaperone activity, its ability to inhibit amyloid fibril formation of alpha-synuclein and the subtle conformational changes at physiologically relevant temperatures may prove useful in understanding the role of HspB2 in normal and disease conditions.
